# The Nexus Between the Big Five Personality Traits Model of the Digital Economy and Blockchain Technology Influencing Organization Psychology

**DOI:** 10.3389/fpsyg.2021.780527

**Published:** 2021-11-25

**Authors:** Yu Dan, Alim Al Ayub Ahmed, Supat Chupradit, Priyanut Wutti Chupradit, Abdelmohsen A. Nassani, Mohamed Haffar

**Affiliations:** ^1^Department of Basic Education, Sichuan Film and Television University, Chengdu, China; ^2^Department of Management, SEGi University Kota Damansara, Kota Damansara, Malaysia; ^3^School of Accounting, Jiujiang University, Jiujiang, China; ^4^Department of Occupational Therapy, Faculty of Associated Medical Sciences, Chiang Mai University, Chiang Mai, Thailand; ^5^Educational Psychology and Guidance, Department of Educational Foundations and Development, Faculty of Education, Chiang Mai University, Chiang Mai, Thailand; ^6^Department of Management, College of Business Administration, King Saud University, Riyadh, Saudi Arabia; ^7^Department of Management, Birmingham Business School, University of Birmingham, Birmingham, United Kingdom

**Keywords:** Big Five model, organizational psychology, block chain technology, digital entrepreneur, digital economy

## Abstract

The basic aim of the study was to understand the role of the Big Five model of personality in predicting emotional intelligence and consequently in triggering the entrepreneurial behavior of the employees. The emotional intelligence of the individuals plays a very important role in decision making, enhancement of quality of living, and many other social realms. Hence, the intelligent use of emotions can make or break an individual’s future considering their attitude toward exploiting the entrepreneurial opportunities available. This study has measured the impact of personality traits on emotional intelligence and EI’s role in digital entrepreneurial behavior. The population used in this study was the middle management employees in the corporate sector of the mainland in China. The sample size taken in this study was 260 and selected through convenient sampling. The data was collected through a structured questionnaire measuring each variable. The data collected was employed to SmartPLS 3.3 for analyzing through structural equation modeling to measure the hypotheses. The study has found the partial effect of the Big Five model of personality on emotional intelligence, which significantly predicted the digital entrepreneurial behavior of the employees. The organizations can use the study findings to anticipate the employees’ possible prospects and endeavors regarding their digital entrepreneurial behaviors.

## Introduction

In the 21st century, the rapidly changing environment’s challenges and transitions in the workplace and society are becoming increasingly common. In this context, organizations are pushed to compete effectively and strive to provide a healthy environment where their employees can flourish. Hence, organizations prefer to hire those employees who can adapt and actively perform in changing environments and eventually enhance corporate performance considering their will to make a decision where necessary and given the authority (Masten, 2014). Moreover, positive relationships of organizational employees improve the well-being of the workplace, which leads to sustainable organizations. Therefore, to identify competent employees at the workplace, organizations seek help from the Big Five personality trait model and their emotional intelligence.

Emotional intelligence has emerged as a potential platform and source for developing sustainable organizations (Di Fabio, 2017). Emotional intelligence encompasses intrapersonal knowledge of an individual, self-motivation, understanding of one’s emotions and management of them, including interpersonal awareness of others’ emotions and respect for their feelings (Chirumbolo et al., 2019). Emotional intelligence can explain some of the remaining variances in predicting work performance and career success, which traditional intelligence has not explained. Employees with high emotional intelligence are better at detecting stress-related feelings and regulating their emotions to decrease it. They can also design strategies to cope with the negative effects of stress. It can be argued that creating a pleasant workplace relational environment would undoubtedly help employees polish their attributes that contribute to their well-being and lead to the development of a sustainable organization through effective decision making.

Personality determines an individual’s behavior and influences their performance at the workplace. The Big Five personality model characterized individual personalities and is globally the most acceptable personality model. Personalities of the individuals have been categorized into five major categories namely: agreeableness, openness, extraversion, neuroticism, and conscientiousness (Teh et al., 2011; Templer, 2012; Kaur and Anand, 2018; Abdellaoui et al., 2019; Dholariya, 2019). It has been acknowledged that key personality traits expressed in the Big Five personality model have a strong association with a wide range of human behaviors (Keefer et al., 2018). Employees with different personality traits behave accordingly; for example, extrovert employees are more active in workplace social networks while conscientious employees have more positive feelings about their workplace (Sutin et al., 2010).

Prior researchers have studied the relationship between Big Five personality traits and emotional intelligence in different contexts (Vesely et al., 2013; Di Fabio and Saklofske, 2014; Luz Martín-Peña et al., 2018; Herrera et al., 2019). These studies conclude that the Big Five personality model includes conscientiousness, agreeableness, openness, neuroticism, and extraversion strongly associated with emotional intelligence.

The main objective of this study is to examine the role of the Big Five model of personality in employees’ emotional intelligence and its consequential role in the digital entrepreneurial behavior of the employees, which contributes to organizational success. This study aims to understand the fundamental questions related to the emotional intelligence of employees, such as:

i What is the role of the Big Five model of personality in emotional intelligence?
ii How does the emotional intelligence of the employees contribute to digital entrepreneurial behavior?

The current study will measure the role of Big Five personality traits in the employees’ emotional intelligence working at the managerial level and how their emotional intelligence factor fosters their attitude toward entrepreneurial behavior. In the next sections of the paper, the literature of concerned variables is reviewed, followed by the methodology and data analysis. The paper is concluded with future recommendations and the limitations of the study.

## Literature Review

### Big Five Personality Traits

**FIGURE 1:**
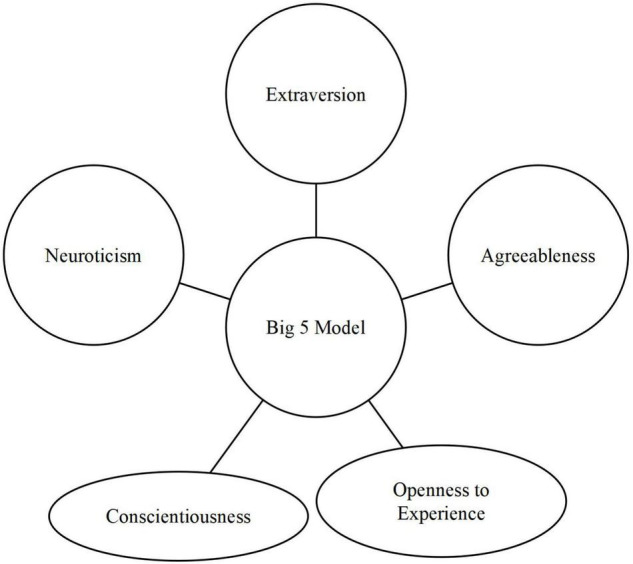
Big Five personality traits model.

**FIGURE 2:**
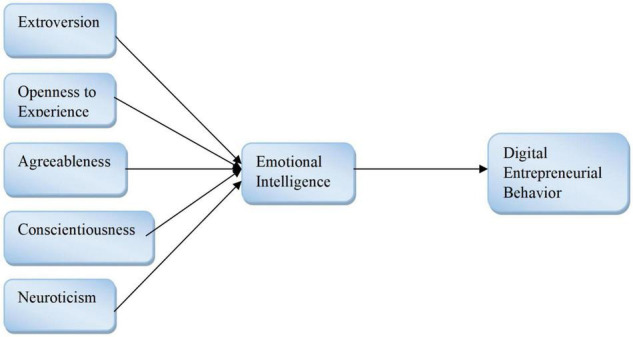
Conceptual model.

Personality traits are characterized as feelings, thoughts, and behaviors that tend to be constant across time and in a relevant context. Goldberg in 1993 proposed the Big Five personality comprehensive framework that encompasses a variety of personality characteristics that is globally accepted to understand the personality of humans (Teh et al., 2011; Baig and Waheed, 2016; Murugesan and Jayavelu, 2017; Kaur and Anand, 2018; Antoñanzas, 2020; Feher and Vernon, 2021). This model consists of five personality traits: consciousness, agreeableness, openness to experience, extraversion, and neuroticism, as given in Figures 1, 2.

Figure 1 explains the five personality traits included in the Big Five models. A brief description of these traits is given below.

#### Conscientiousness

Conscientiousness is defined as an individual’s willingness to accomplish a specific task, that is, to be dependent and persistent until the project is done (Murugesan and Jayavelu, 2017). Conscientious employees have the characteristics such as competence, organization, willingness, fight achievement, consideration, and self-discipline. Individuals with a high level of conscientiousness are more capable of weighing the pros and cons of a particular scenario (El Othman et al., 2020).

#### Agreeableness

Agreeableness describes nurturance, altruism, care, and emotional support, and it is also linked to being cooperative, trustful, tolerant, and forgiving (Digman, 2003). Friendly individuals have an emotional concern for the well-being of others, treat others with consideration for their rights and preferences, and usually have favorable opinions about others (Soto, 2019). Agreeableness positively impacts the intuitive and reliant decision-making styles (El Othman et al., 2020). Employees with a low level of agreeableness are aggressive, oppositional, manipulative, callous, and strong-willed (Shehzad et al., 2020, 2021). Agreeable employees are more motivated to establish an interpersonal connection which contributes to a higher level of well-being and satisfaction (Aydogmus et al., 2015).

#### Openness to Experience

Openness to experience is defined as more adventurous and open to experiencing new things, high on intellect and related to regularities that individuals find as an indicator of intelligence in others’ lives. It is mostly linked to political ideas, cultural behavior, intelligence, and creativity (Schwaba et al., 2018). Openness to experience is associated with divergent thinking, intelligence, imagination, originality, and broad-mindedness (Cornwell et al., 2020). Employees with higher scores in this attribute show a need for diversity and unconventional values (McCrae and Costa, 2013).

#### Extraversion

Extraversion is associated with going out, socializing, and being friendly, talkative, and energetic (Arpaci et al., 2018). Extrovert individuals have more positive autobiographical experiences than neurotic individuals (Denkova et al., 2012). Individuals with a high level of extraversion traits are more capable of weighing the pros and cons of a particular scenario (El Othman et al., 2020). Extraversion has been identified as the main indicator of social behavior and had a favorable impact on spontaneous decision-making style (Soto and Tackett, 2015; Sarfraz et al., 2021). Highly extraverted employees are more friendly, sociable, outgoing, and they can understand their own and other’s employees’ emotions as compared to low extroversion Nawi (Hudani et al., 2012).

#### Neuroticism

Neuroticism is a personality trait that naturally reflects variations in positive and negative emotions (Soto, 2019). Anxiety, sadness, poor self-esteem, impulsivity, and mood fluctuations are mostly common traits among highly neurotic individuals. Therefore, neuroticism scores are predicted to be low in positive output behaviors (Murugesan and Jayavelu, 2017). Individuals having high neuroticism levels are more likely to adopt maladaptive techniques to control their emotions and are less likely to participate in reappraisal, and have more negative moods (Yoon and Barker Steege, 2013). Neurotic employees experience more negative life events, and such employees are more furious, depressed, embarrassed, and worried and more focused on the negativity around them (Magnus et al., 1993; Tong, 2010; Blackwell et al., 2017). These employees have a hard time expressing their feelings and understanding the behavior of the other employees in the organization (Aydogmus et al., 2015).

### Emotional Intelligence

Emotional intelligence is defined as an individual’s ability to access and describe his own and others’ emotions accurately. To retrieve and generate feelings in the thinking process; more inclined to control and apply emotions in problem-solving processes (Salovey and Sluyter, 1997). In simple words, emotional intelligence encompasses both intrapersonal knowledge of oneself, self-motivation, awareness of one’s own emotions and managing these emotions not only for themselves but also understanding and deciding to respect others feelings (Zampetakis et al., 2009; Aydogmus, 2016; Di Fabio and Kenny, 2016; Devries et al., 2018; Liébana-Presa et al., 2020). These characteristics of intra-interpersonal awareness enable one to acquire an in-depth understanding of relationships (Di Fabio and Kenny, 2016).

Emotional intelligence can explain some of the remaining variances in predicting work performance and career success which has not been explained by traditional intelligence. Employees with a high level of emotional intelligence are more likely to be satisfied with their lives, have stronger personal and social connections, and achieve professional success (Amdurer et al., 2014; Sony and Mekoth, 2016). Employee emotional intelligence is significantly linked with organizational success, such as performance and organizational commitment (Kafetsios and Zampetakis, 2008).

### Digital Entrepreneurship

Digital entrepreneurship has been defined as the new start-up of a business to take risks in the hope of earning profits. In the last decade, physical things have been digitalized using social media, mobile services, clouds, big data, robotics, etc. (Elia et al., 2020). It has also helped the entrepreneurs to partner, collaborate, meet the demands, and develop new solutions and standards. This has given a new direction to entrepreneurial minds to exploit maximum opportunities with minimum resources (Obschonka et al., 2017; Elia et al., 2020). Previously many studies have been carried out from different perspectives to understand what personal and behavioral intentions of entrepreneurs distinguish them from ordinary people. Some factors that separate traditional entrepreneurs from digital entrepreneurs are easiness of entry, easiness of doing business, digital inventory, digital infrastructure, digital tools, and digital workplace (Taleghani et al., 2013; Elia et al., 2020).

Digital technologies have been a vital component in the start-ups of online businesses. Hence, it can be said that these novel technologies are the enablers for digital entrepreneurship. This makes the use of this platform for reaching new ventures and stakeholders like Netflix, meeting multidimensional demands like Uber, getting paid for work done online like Upwork and Fiverr (Elia et al., 2020). Using the Big Five model of personality to measure digital entrepreneurial intentions is suitable because previously, many studies have been conducted where Big Five models have yielded surprisingly accurate results (Back et al., 2010; Boyd and Pennebaker, 2016; Obschonka et al., 2017).

### Emotional Intelligence and Big Five Personality Traits

Emotional intelligence is considered a predictor of the Big Five personality trait model (Avsec et al., 2009). The employees with higher scores on personality traits and emotional intelligence are more related to better task performance and managing emotions with others (O’Boyle et al., 2011). Prior literature reveals a strong association between emotional intelligence and Big Five-factor personality traits (Avsec et al., 2009; Aydogmus, 2016; Alghamdi et al., 2017; Antoñanzas, 2020; El Othman et al., 2020; Feher and Vernon, 2021).

### Conscientiousness and Emotional Intelligence

Conscientiousness is the most important factor in emotional intelligence (Hudani et al., 2012; Aydogmus et al., 2015). Antoñanzas (2020) argued that conscientiousness has a positive correlation with emotional intelligence. According to the findings of Day et al. (2005), a strong relationship exists between emotional intelligence and conscientiousness. Individuals with a high level of conscientiousness are more capable of weighing the pros and cons of a particular scenario (El Othman et al., 2020). The previously mentioned literature helps to develop the following hypothesis as follows:

***H_1_:*** *Conscientiousness trait has a positive relationship with emotional intelligence.*

### Agreeableness and Emotional Intelligence

Agreeable employees are more motivated to establish an interpersonal connection, which contributes to higher well-being and satisfaction (Aydogmus et al., 2015). Agreeableness captures differences in respect, love, and acceptance of others. Friendly individuals have an emotional concern for the well-being of others, treat others with consideration for their rights and preferences, and usually have favorable opinions about others (Soto, 2019). Agreeableness and emotional intelligence are significantly correlated with one another, highly agreeable employees are warm and are sensitive to others’ wishes (Aydogmus, 2016; Jonason et al., 2017; Urquijo et al., 2019). The previously mentioned literature helps to develop the following hypothesis as follows:

***H_2_:*** *The agreeableness trait has a positive relationship with emotional intelligence.*

### Openness and Emotional Intelligence

Openness to experience is defined as the readiness of an individual to try out new things, high intellect, and related to regularities that individuals find as an indicator of intelligence in others’ lives. It is mostly linked to political ideas, cultural behavior, intelligence, and creativity (Schwaba et al., 2018). Employees high in openness to experience are more inventive and aggressive in their search for new opportunities (Strik et al., 2019). Openness to experience significantly affects employees’ performance at the workplace, and people with high emotional intelligence are anticipated to achieve more success and contribute considerably to organizational performance (Carmeli et al., 2009). The previously mentioned literature helps to develop the following hypothesis as follows:

***H_3_:*** *Openness to experience has a positive relationship with emotional intelligence.*

### Extraversion and Emotional Intelligence

Extraversion has been identified as the main indicator of social behavior and had a favorable impact on the spontaneous style of decision making (DeYoung et al., 2007). Extrovert individuals have positive autobiographical experiences as compared to neurotic individuals (Denkova et al., 2012). High extroversion employees are more friendly, sociable, outgoing, and they can understand their own and other’s employees’ emotions compared to low extroversion (Hudani et al., 2012). According to Day et al. (2005), a strong relationship exists between the people high on extraversion and more prone to making emotionally intelligent decisions. Based on the above literature, we propose our next hypothesis:

***H_4_:*** *Extraversion trait has a positive relationship with emotional intelligence.*

### Neuroticism and Emotional Intelligence

Neurotic individuals are worried, depressed, and vulnerable, and they have a pessimistic outlook on life therefore, neuroticism scores are predicted to be low (Murugesan and Jayavelu, 2017). These employees have a hard time expressing their feelings and understanding the other employees’ behavior in the organization (Aydogmus, 2016). Individuals with high neuroticism levels are more likely to adopt maladaptive techniques to control their emotions and are less likely to participate in reassessment and usually have more negative moods (Yoon and Barker Steege, 2013). Previous studies have described neuroticism as having a negative relationship with emotional intelligence (Sandhu et al., 2009). Neuroticism makes the least contribution to emotional intelligence and has no significant impact on emotional intelligence (Alghamdi et al., 2017). Based on the above discussion, we proposed our hypothesis as follows:

***H_5_:*** *Neuroticism trait has a negative relationship with emotional intelligence.*

### Emotional Intelligence and Digital Entrepreneurial Behavior

The characteristic of an individual to understand the emotional behavior of others and their own self has been a key reason for success of such individuals (Di Fabio and Kenny, 2016). Previous research has examined that emotional intelligence is a strong forecaster of entrepreneurial behavior. However, personality traits have been an important constituent of entrepreneurial studies (Obschonka et al., 2017; Alexandru et al., 2019). An individual’s confidence in his abilities to be successful in his tasks and intentions has also been a vital factor for their entrepreneurial achievements. Hence, emotional intelligence is predicted to make a significant role in digital entrepreneurial behavior.

***H_6_:*** *Emotional intelligence has a positive relationship with digital entrepreneurial behavior.*

## Research Methods

**FIGURE 3:**
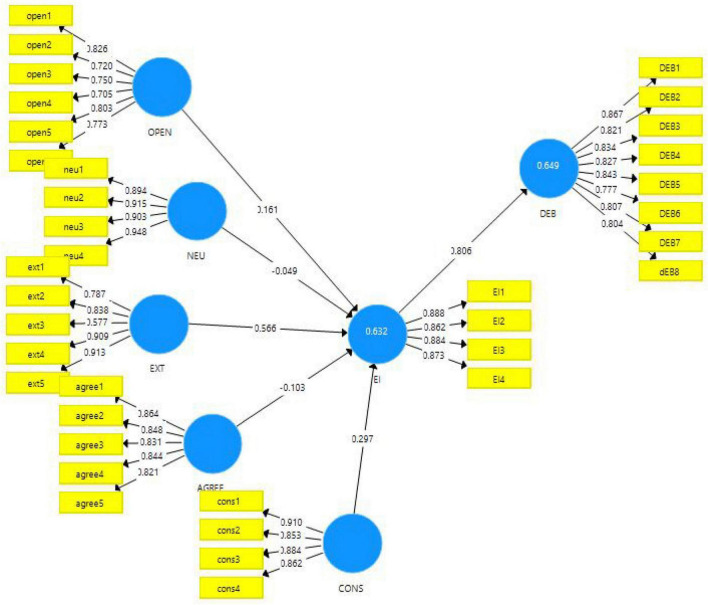
Measurement model algorithm outcomes.

**FIGURE 4:**
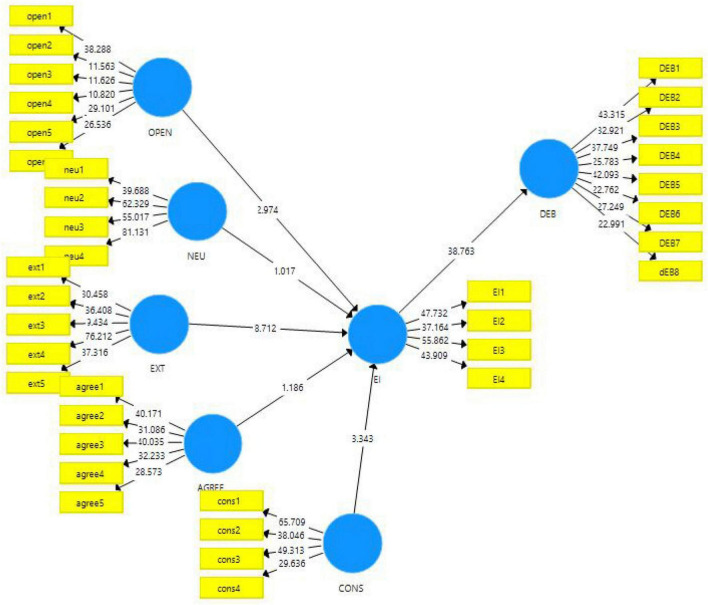
PLS-structural model algorithm outcomes.

The current study follows the quantitative approach with the survey method for data collection. Since this study is about measuring the effects of personality traits mentioned in the Big Five model of personality on emotional intelligence and consequently on digital entrepreneurship behavior, the philosophy followed is post-positivist. The theories have been devised based on the literature, and hypotheses are formed to reach certain conclusions. Therefore, a deductive approach of research is followed. Further, the hypotheses were tested for their approval and rejection based on the data collected from the sample selected. The population frame used in this study was made up of student employees at middle-level management in the mainland in China. The data obtained were checked for reliability and validities and then further run on SmartPLS software 3.3. The results obtained were used to reach the acceptance and rejection of the hypotheses of the study, as given in Figures 3, 4.

The sample was selected through convenience sampling because reaching out to all the population was not feasible considering a large strength of employees in the corporate sector. Hence, the sample size taken in this study was 260 which is considered a good sample size. We distributed the questionnaire to 292 employees and 260 were reliable to estimate the Structural Equation Modeling (SEM) analysis. The rest were screened out due to maintaining the reliability of the analysis. The unit of analysis is the employees from the corporate sector in the mainland in China. The data collection is done through a structured questionnaire and a survey technique was employed. The respondents were informed about the survey beforehand, appointments were taken, and the questionnaires were filled right away to avoid ambiguity in understanding items. All the measurements are considered from previously well-known and accepted studies. The data collected from the sample was then used in the SmartPLS software for the structural equation modeling.

### Measurement Scale

This study considered measurement scales from earlier research for all the constructs to measure the concerned variables in the model. Overall, 24 items were used to measure the Big Five personality traits and emotional intelligence. The author considered a 24-item scale for each Big Five personality trait from Teh et al. (2011) and a four-item short-scale form (Marchena-Giráldez et al., 2021) to measure the emotional intelligence in this study and digital entrepreneurial behavior from Davidson and Vaast (2010). All the items were measured through a 5-point Likert Scale from (1 to 5) Strongly Agree to Strongly Disagree.

## Analysis and Results

This analysis is based on the SEM, a two-stage estimation in Smart Partial Least Square (SmartPLS). The SEM analysis has two sequential steps, including estimating the measurement model and structural model. Both of these steps share different purposes. Measurement model estimation aims to estimate the reliability and validity of constructs and the items. This step also usually has the measurements of convergent validity and discriminant validity. The reliability, convergent validity, and discriminant validity are measured through Cronbach alpha and construct’s reliability, AVE values, factor loadings, Fornell and Larcker ratio, and HTMT ratio. This step helps the estimation process to analyze the reliability of constructs that are in the research model. The summary of demography is mentioned in Table 1.

**TABLE 1:** Demographic summary.

	Frequency	%
Gender		
Male	135	51.92
Female	125	48.07
Do you think personality traits are important?		
Yes	190	73.07
No	70	26.92
Which personality trait is more important in the Big Five?		
Consciousness	60	23.07
Agreeableness	65	25.00
Openness	65	25.00
Extraversion	10	3.86
Neuroticism	60	23.07
Does the big personality model describe individual behavior?		
Yes	225	86.53
No	35	13.46
Do you think, personality can alter emotional intelligence?		
Yes	200	76.92
No	60	23.07

*N = 260.*

Table 1 illustrates the summary of respondents in this research. The questionnaire of the research included four demographical questions. Overall, the outcomes of empirical details demonstrated that males and females have equally participated in the survey thus, there are no such biases in the outcome of the research, which are 48 and 52%, respectively. Moreover, around 73% of respondents believe that the Big Five personality traits are important to understand the personality and its effect on emotional intelligence.

The first stage of SEM analysis is the measurement model. This study considered the Cronbach alpha and Construct Reliability (CR) to estimate the reliability of all constructs in the model. All the values of Cronbach alpha and CR are above the threshold of 0.70 (Huo et al., 2020; Lia et al., 2020). Thus the reliability of all six constructs is satisfied thus reliable to use. The factor loadings measure the items or individual measurements reliability to measure the distinct construct, and it must be greater than 0.70 (Hair et al., 2017). Thus all items of each contract are above the threshold thus, items are reliable. Afterward, the AVE values are also above the threshold of 0.50 (Hair et al., 2017) and all values above 0.50 demonstrated the discriminant validity. Thus, the convergent validity is maintained, and all constructs and their items are reliable and converted to measure the construct. All the outcome coefficients are illustrated in Table 2.

**TABLE 2:** Reliabilities and variance extracted.

Constructs	Code	FD	α	CR	AVE
Conscientiousness			0.900	0.931	0.770
	cons1	0.910			
	cons2	0.853			
	cons3	0.884			
	cons4	0.862			
Agreeableness			0.897	0.924	0.708
	agree1	0.864			
	agree2	0.848			
	agree3	0.831			
	agree4	0.844			
	agree5	0.821			
Openness to experience			0.872	0.893	0.584
	open1	0.826			
	open2	0.720			
	open3	0.750			
	open4	0.705			
	open5	0.803			
	open6	0.773			
Extraversion			0.867	0.906	0.663
	ext1	0.787			
	ext2	0.838			
	ext3	0.577			
	ext4	0.909			
	ext5	0.913			
Neuroticism			0.935	0.954	0.837
	neu1	0.894			
	neu2	0.915			
	neu3	0.903			
	neu4	0.948			
Emotional intelligence			0.900	0.930	0.769
	EI1	0.888			
	EI2	0.862			
	EI3	0.884			
	EI4	0.873			
Digital entrepreneurial behavior			0.932	0.944	0.677
	DEB1	0.867			
	DEB2	0.821			
	DEB3	0.834			
	DEB4	0.827			
	DEB5	0.843			
	DEB6	0.777			
	DEB7	0.807			
	DEB8	0.804			

*N = 260.*

However, respondents believe that all big traits are equally important, but the extraversion trait is found less important based on the respondent’s details. Afterward, more than 86% of people believe that these five traits describe individuals’ overall behavior, and 75% respond that these traits can alter individuals’ emotional intelligence. Secondly, the structural model assessment estimated the causal relationship between the variables in this case; this stage will produce the statistical significance of paths or relationship between the Big Five personality traits and emotional intelligence. This study considered SmartPLS 3.3.3. on 5000 sub-sample in algorithm and bootstrapping estimation stages.

The discriminant validity is measured through the Fornell and Larcker criterion of correlation and the HTMT (Heterotrait-Monotrait) Ratio (Hair et al., 2017). Both measures are used to measure the discriminant validity of the constructs. These two tests are used to measure if there is any case of multicollinearity. If the values of the HTMT ration (according to Franke and Sarstedt, 2019) are below 0.90, these results indicate that the variables in the study are discriminantly valid and do not have any impact on each other. Since this study showed all the diagonal values above the below values, the discriminant validity is maintained, and it showed that there is no such issue of higher correlation. It implies that items of concerning variables cannot discriminate with each other thus are unable to satisfy the discriminant validity. The results for Fornell and Larcker criterion were illustrated in Tables 3, 4.

**TABLE 3:** Fornell and Larcker criterion.

	AGREE	CONS	DEB	EI	EXT	NEU	OPEN
AGREE	0.842						
CONS	0.881	0.878					
DEB	0.648	0.665	0.823				
EI	0.564	0.605	0.806	0.877			
EXT	0.597	0.600	0.879	0.764	0.814		
NEU	0.209	0.190	0.212	0.286	0.387	0.915	

*N = 260.*

**TABLE 4:** HTMT ratio.

	AGREE	CONS	DEB	EI	EXT	NEU	OPEN
AGREE							
CONS	0.909						
DEB	0.710	0.721					
EI	0.623	0.669	0.874				
EXT	0.666	0.669	0.654	0.855			
NEU	0.228	0.207	0.229	0.311	0.680		
OPEN	0.483	0.418	0.589	0.549	0.480	0.686	

*N = 260.*

The second part of the analysis measured the structural model assessment. The structural model assessment is related to the understanding of the casual relationship. Structural models are validated by computing beta (β), *R*^2^, and corresponding *t*-values using a bootstrapping methodology based on a 5000 resampling. The structural model assessment results are illustrated in Table 5. Start with the first hypothesis (H_1_) the agreeableness does not predict the emotional intelligence with *t*−*statistic* = 1.186thus H_1_ confirmed statistically insignificant relationship. Secondly, the second trait of the Big Five models is consciousness. Consciousness meaningfully predicts emotional intelligence as consciousness has a positive significant impact on emotional intelligence under *t*−*statistic* = 3.343:*p*−*value* = 0.000 therefore H_2_ is also confirmed. Thirdly, the extraversion trait also meaningfully predicts the emotional intelligence among employees as the *p*-values and *t*-statistics imply a significant relationship between these constructs as *t*−*statistic* = 8.712:*p*−*value* = 0.000 so H_3_ is also accepted. Fourth, the H_4_ was rejected since it did not demonstrate that neuroticism does not have a significant positive impact on emotional intelligence as *t*−*statistic* = 1.017:*p*−*value* = 0.310. Finally, the last trait, openness also demonstrated a positive and significant impact on emotional intelligence as *t*−*statistic* =  2.974:*p*−*value* = 0.003. Emotional intelligence also showed the most powerful impact on digital entrepreneurial behavior *t*−*statistic* = 38.763:*p*−*value* = 0.000 thus accepting the mediating role of emotional intelligence H_6_.

**TABLE 5:** Direct effects.

H.	Paths	O	M	STDEV	*t*-Stats	*p*-Values	*R* ^2^
*H1*	AG → EI	−0.103	−0.099	0.086	1.186	0.236	0.624
*H2*	CON → EI	0.297	0.297	0.089	3.343	0.001	
*H3*	EX → EI	0.566	0.563	0.065	8.712	0.000	
*H4*	NE → EI	−0.049	−0.047	0.048	1.017	0.310	
*H5*	OP → EI	0.161	0.161	0.054	2.974	0.003	
*H6*	EI → DEB	0.806	0.808	0.021	38.763	0.000	0.648

*N = 260. H., hypothesis; O, original sample; M, sample mean, STDEV, standard deviation.*

## Discussion

Big Five personality traits are a source of emotional intelligence these days (Di Fabio and Saklofske, 2021). This study has explored the exogenous effect of the Big Five personality traits model on employees’ emotional intelligence in Chinese settings. The Five – factors personality theory provides a straightforward framework for comprehending others and enhancing relationships by understanding why individuals behave the way people do. Several psychologists now consider that the five personality traits are biologically based as well as universally accepted. These personal attributes represent the most significant elements that define our social environment. The discussion part compares and contrasts the findings of the current study with the earlier literature. Therefore, the below discussion emphasizes the current findings of research with previous literature.

This study considered the parallel relationship from Kappagoda (2013). The study’s findings and interpretations are based on the measurement and structural models obtained from the structural equation modeling. As a preliminary step, the data obtained from the respondents were checked for reliability. The reliabilities obtained in this study were above 0.8, and the AVE for the variables was above the cut-off value of 0.5 (Sarstedt et al., 2019). Similarly, the Fornell and Larcker criterion and HTMT ratios gave the values that meet the acceptability criteria for these two tests (see Tables 3, 4). The hypotheses of the study developed from the literature were measured using the path model. The results obtained can be seen in Table 5.

Overall, this research demonstrated that out of five personality traits, three personality traits demonstrated a positive association with emotional intelligence, however, previously it has been found that emotional intelligence also meaningfully correlated with the Big Five personality traits (Avsec et al., 2009; Aydogmus, 2016; Boyd and Pennebaker, 2016; Obschonka et al., 2017; Antoñanzas, 2020; El Othman et al., 2020). Day et al. (2005) concluded that emotional intelligence has a significant association with extraversion, openness, and agreeableness in particular, and no significant association was found between neuroticism and consciousness. The previous research findings strongly defend and strengthen our research findings because few personality traits may not have a significant relationship with emotional intelligence as in current authors found a non-significant association of agreeableness and neuroticism with emotional intelligence. This factor may be prevailed due to different cultural aspects, values, and norms thus produced different results.

According to the best of our knowledge, this study is the first to consider the direct effect of the Big Five personality traits individually on emotional intelligence as mediating variable and digital entrepreneurship as the dependent variable. This study investigated the direct effect of the Big Five personality traits (extraversion, openness, agreeableness, neuroticism, and consciousness) on employees’ emotional intelligence in Chinese settings. If seen individually, agreeableness could not find significant results in this study which is in contradiction with the previous studies (Aydogmus, 2016; Jonason et al., 2017; Soto, 2019; Urquijo et al., 2019) this is because people higher on agreeableness tend to please others as much as they can which drives them away from their emotional intelligence (Obschonka et al., 2017). The first rejected hypothesis implies that emotionally less intelligent employees can agree with people’s opinions on different points and fail to manage their own emotions compared to those who are high on the power of acceptance and agreeableness. As for conscientiousness, it has found significant results regarding their positive impact on emotional intelligence, which is in adherence with the studies conducted in the past (Day et al., 2005; Hudani et al., 2012; Aydogmus et al., 2015; Antoñanzas, 2020; El Othman et al., 2020). Secondly, the conscientiousness of employees tend to have a thoughtful mind, mindful and much organized than unconscious employees son the workplace. Therefore, people those have consciousness personality trait often are emotionally intelligent and protectively manage their work activities.

Furthermore, extraversion also found a significant positive impact on emotional intelligence, supported by the past findings of Day et al. (2005). Extraversion may not influence emotional intelligence in Chinese settings. It may be because of the introverted personalities of Chinese employees. This fact is also demonstrated in demographic details that extraversion may not be an important personality trait according to respondents. Neuroticism in this study could not find significance in emotional intelligence. These findings follow (Sandhu et al., 2009; Alghamdi et al., 2017) because negative vibes do not add to the emotional intelligence but rather wear off. The positive association of neuroticism with emotional intelligence describes that Chinese employees are experiencing a lot of stress. Lastly, the finding regarding the openness to experience have also been aligned with the past research of Carmeli et al. (2009) who found that individuals with high openness to experience are more prone to emotional intelligence. Finally, the openness also demonstrated a positive association that meaningfully explains that employees are open to learning new skills and competence are emotionally intelligent. Therefore, employees with these traits are emotionally intelligent and effective at the workplace (Kappagoda, 2013; Antoñanzas, 2021; Di Fabio and Saklofske, 2021). The last hypothesis of the study about emotional intelligence playing a role in digital entrepreneurial behavior has been in line with the past researches (Obschonka et al., 2017; Elia et al., 2020) who found that emotional intelligence has been a key contributor to the entrepreneurial activities and behaviors of individuals. This is because the individuals higher on EI tend to appear stronger for decision making and hence contribute to the entrepreneurial activities. The ultimate interest of this study was to check the role of emotional intelligence in bridging the relationship of personality traits and the digital entrepreneurial behavior. There have been very less studies considering the changing demands of the entrepreneurship taking into account the emotional intelligence. Hence, this study has tried to empirically check these relationships among the variables of interest. It has been found that emotional intelligence very significantly and strongly predicts the digital entrepreneurial behaviors. The findings of this study endorse the previous studies (Zampetakis et al., 2009; Taleghani et al., 2013; Obschonka et al., 2017).

## Conclusion

The Big Five personality model has been found to have great significance in improving emotional intelligence of individuals. This study has investigated the relationship between the Big Five personality traits and the emotional intelligence and consequently on the entrepreneurial behaviors among employees in China. The study has found a partial role of Big Five model of personality on emotional intelligence not finding significant results for agreeableness and neuroticism. Moreover, emotional intelligence has positively and significantly predicted the digital entrepreneurial behavior of employees in China. The results signify the importance of exploiting opportunities available to the middle-level employees in progressing in their fields. These findings are important for human resource specialists and other top management stakeholders to understand the employee’s behavior. The results suggest that employees in the China should be given opportunities according to their job descriptions to exploit their full potential. Moreover, it also highlights the potential importance of personality traits in terms of emotional intelligence because it supports employees in effectively managing work activities at the workplace.

### Limitations and Future Research

This research has few research limitations. Firstly, this study is a cross-sectional study; thus, more research is required in longitudinal nature in the current scenario to collect more data on the said variables and produce more comprehensive results in understanding employees’ aptitude in organizations regarding their emotional intelligence and prospects of entrepreneurship. Moreover, this research is conducted in China thus, a clear representation of Chinese cultural settings can be observed in the research outcomes. But these findings are based on a single culture (Chinese), and country constrained; therefore, more research is required to generalize these findings in different emerging or developed countries like Pakistan or the United States. Moreover, moderating variables such as organizational support can be used in future studies to understand better the model proposed in this study.

## Data Availability Statement

The original contributions presented in the study are included in the article/supplementary material, further inquiries can be directed to the corresponding author.

## Ethics Statement

Ethical approval for this study and written informed consent from the participants of the study were not required following local legislation and national guidelines.

## Author Contributions

YD and AA: initial draft and methods. SC and PC: revision and supervision. AN and MH: analysis and interpretation. All authors contributed to the article and approved the submitted version.

## Conflict of Interest

The authors declare that the research was conducted in the absence of any commercial or financial relationships that could be construed as a potential conflict of interest.

## Publisher’s Note

All claims expressed in this article are solely those of the authors and do not necessarily represent those of their affiliated organizations, or those of the publisher, the editors and the reviewers. Any product that may be evaluated in this article, or claim that may be made by its manufacturer, is not guaranteed or endorsed by the publisher.
